# Prospective Home-use Study on Non-invasive Neuromodulation Therapy for Essential Tremor

**DOI:** 10.5334/tohm.59

**Published:** 2020-08-14

**Authors:** Stuart H. Isaacson, Elizabeth Peckham, Winona Tse, Olga Waln, Christopher Way, Melita T. Petrossian, Nabila Dahodwala, Michael J. Soileau, Mark Lew, Cameron Dietiker, Nijee Luthra, Pinky Agarwal, Rohit Dhall, John Morgan, Nicole Calakos, Theresa A. Zesiewicz, Ejaz A. Shamim, Rajeev Kumar, Peter LeWitt, Holly A. Shill, Adam Simmons, Fernando L. Pagan, Pravin Khemani, Jessica Tate, Brian Maddux, Lan Luo, William Ondo, Mark Hallett, Apoorva Rajagopal, Paula Chidester, Kathryn H. Rosenbluth, Scott L. Delp, Rajesh Pahwa

**Affiliations:** 1 Parkinson’s Disease and Movement Disorders of Boca Raton, Boca Raton, FL, US; 2 Central Texas Neurology Consultants, Round Rock, TX, US; 3 Mount Sinai Hospital, Department of Neurology, New York, NY, US; 4 Houston Methodist, Department of Neurology, Houston, TX, US; 5 Parkinson’s Institute and Clinical Center, Mountain View, CA, US; 6 Pacific Neuroscience Institute, Pacific Movement Disorders Center, Santa Monica, CA, US; 7 University of Pennsylvania, Department of Neurology, Philadelphia, PA, US; 8 Texas Movement Disorders Specialists, Georgetown, TX, US; 9 University of Southern California, Department of Neurology, Los Angeles, CA, US; 10 University of California San Francisco, Movement Disorder and Neuromodulation Center, San Francisco, CA, US; 11 EvergreenHealth, Department of Neurology, Kirkland, WA, US; 12 University of Arkansas for Medical Sciences, Department of Neurology, Little Rock, AR, US; 13 Augusta University, Department of Neurology, Augusta, GA, US; 14 Duke University School of Medicine, Department of Neurology, Durham, NC, US; 15 University of South Florida Health, Department of Neurology, Tampa, FL, US; 16 Kaiser Permanente MidAtlantic States, Department of Neurology, MidAtlantic Permanente Research Institute, Largo, MD, US; 17 Rocky Mountain Movement Disorders Center, Englewood, CO, US; 18 Henry Ford Health System, Department of Neurology, West Bloomfield, MI, US; 19 Barrow Neurological Institute, Department of Neurology, Phoenix, AZ, US; 20 Hospital for Special Care, Department of Research, New Britain, CT, US; 21 Georgetown University Medical Center, Department of Neurology, Washington DC, US; 22 Swedish Neuroscience Institute, Department of Neurology, Seattle, WA, US; 23 Wake Forest Baptist Health, Department of Neurology, Winston-Salem, NC, US; 24 Riverhills Neuroscience, Cincinnati, OH, US; 25 Beth Israel Deaconess Medical Center, Harvard Medical School, Department of Neurology, Boston, MA, US; 26 National Institute of Neurological Disorders and Stroke, Human Motor Control Section, Bethesda, MD, US; 27 Cala Health, Burlingame, CA, US; 28 Stanford University, Department of Bioengineering, Stanford, CA, US; 29 University of Kansas Medical Center, Department of Neurology, Kansas City, KS, US

**Keywords:** clinical trials, tremor, neuromodulation, stimulation, non-invasive

## Abstract

**Highlights:**

This prospective study is one of the largest clinical trials in essential tremor to date. Study findings suggest that individualized non-invasive neuromodulation therapy used repeatedly at home over three months results in safe and effective hand tremor reduction and improves quality of life for many essential tremor patients.

**Background:**

Two previous randomized, controlled, single-session trials demonstrated efficacy of non-invasive neuromodulation therapy targeting the median and radial nerves for reducing hand tremor. This current study evaluated efficacy and safety of the therapy over three months of repeated home use.

**Methods:**

This was a prospective, open-label, post-clearance, single-arm study with 263 patients enrolled across 26 sites. Patients were instructed to use the therapy twice daily for three months. Pre-specified co-primary endpoints were improvements on clinician-rated Tremor Research Group Essential Tremor Rating Assessment Scale (TETRAS) and patient-rated Bain & Findley Activities of Daily Living (BF-ADL) dominant hand scores. Other endpoints included improvement in the tremor power detected by an accelerometer on the therapeutic device, Clinical and Patient Global Impression scores (CGI-I, PGI-I), and Quality of Life in Essential Tremor (QUEST) survey.

**Results:**

205 patients completed the study. The co-primary endpoints were met (p≪0.0001), with 62% (TETRAS) and 68% (BF-ADL) of ‘severe’ or ‘moderate’ patients improving to ‘mild’ or ‘slight’. Clinicians (CGI-I) reported improvement in 68% of patients, 60% (PGI-I) of patients reported improvement, and QUEST improved (p = 0.0019). Wrist-worn accelerometer recordings before and after 21,806 therapy sessions showed that 92% of patients improved, and 54% of patients experienced ≥50% improvement in tremor power. Device-related adverse events (e.g., wrist discomfort, skin irritation, pain) occurred in 18% of patients. No device-related serious adverse events were reported.

**Discussion:**

This study suggests that non-invasive neuromodulation therapy used repeatedly at home over three months results in safe and effective hand tremor reduction in many essential tremor patients.

## Introduction

Essential tremor (ET) is one of the most common movement disorders [1]. Upper limbs are affected in virtually all ET patients, and other regions (e.g., head, voice, and lower limbs) are affected in some patients [2,3]. ET can be physically, psychologically, and socially detrimental, and reduce the quality of life for patients [4,5,6,7,8,9]. The mechanisms of ET are not completely understood, but studies comparing neural activity, brain imaging, and electromyography data between ET patients and healthy adults suggest that ET is caused by rhythmic signaling within a central tremor neural network involving the ventral intermediate nucleus (VIM) of the thalamus [10,11,12,13,14,15,16].

Current pharmacotherapy options for ET include the use of nonselective β-blockers (propranolol) and anticonvulsants (primidone) as first-line treatments, and topiramate, benzodiazepines, gabapentin, zonisamide, and pregabalin as second-line treatments, but patient responses to these medications are variable [17,18,19,20,21,22]. For patients who do not respond to medications, current alternative options are invasive neurosurgical procedures, including VIM deep brain stimulation (DBS), or magnetic resonance-guided focused ultrasound (MRgFUS) VIM thalamotomy [17,23]. These second-line options, while effective for many, carry the significant safety risks and expenses associated with invasive procedures [24,25].

Previous research demonstrating that electrical stimulation of peripheral nerves at the wrist evoked activity within the VIM and other regions of the central tremor network led to the development of a non-invasive neuromodulation therapy called Transcutaneous Afferent Patterned Stimulation (TAPS) [26,27]. TAPS consists of bursts of non-invasive electrical stimulation alternating between the median and radial nerves at the wrist at a frequency tuned to an individual patient’s tremor. Two sham-controlled, randomized, single-session studies have shown TAPS to be a safe and effective symptomatic ET treatment [28,29], leading to United States Food and Drug Administration (FDA) clearance [30,31]. However, it is unknown how these single-session findings on TAPS safety and efficacy translate to longer-term efficacy as the therapy is used at home.

The goal of this study was to expand understanding of efficacy and safety of TAPS from usage in a single session to three months of repeated use. Efficacy was measured using clinical gold standard measurements, patient-reported outcomes, and objective kinematic tremor physiology endpoints. The study was run without a blinded sham arm due to the challenge of mimicking the sensation of stimulation or otherwise maintaining blind with an at-home device over three months of repeated use.

## Methods

### Study design and patient population

**Figure 1:**
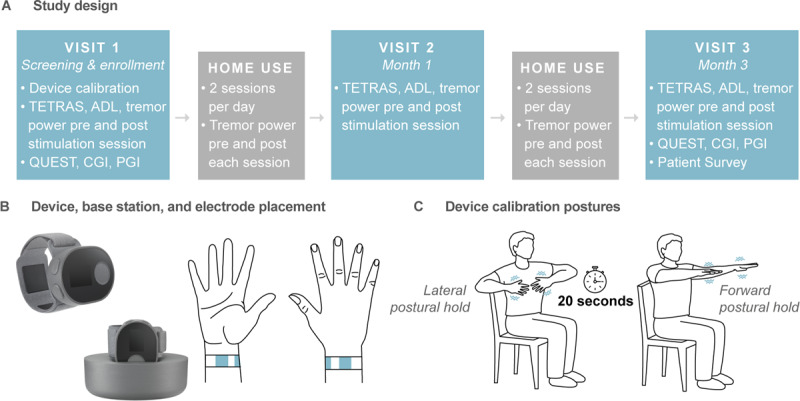
**Study design, therapeutic device, and calibration postures. (A)** The study included 3 in-clinic visits over 3 months with interim prescribed twice-daily home-use of therapy. **(B)** The wrist-worn device consisted of a stimulator, detachable band, and base station. The stimulator applied the stimulation pattern to the band and had an onboard triaxial accelerometer to measure tremor. The band contained two working electrodes positioned over the median and radial nerves and a counter-electrode positioned on the dorsal side of the wrist. The base station streamed accelerometer and usage data daily and charged the device. **(C)** Patients performed either a lateral or forward postural hold for device calibration and for tremor measurement pre- and post-stimulation.

This study was a prospective, multi-center, single-arm, open-label clinical trial to evaluate the safety and efficacy of TAPS therapy over a three-month period. The therapy was delivered with an FDA-cleared wrist-worn neuromodulation device (Cala Health, Inc.; Burlingame, CA, USA). The study was registered as clinical trial (NCT03597100, clinicaltrials.gov) entitled Prospective Study for Symptomatic Relief of ET with Cala Therapy (PROSPECT). The study included three in-clinic visits: Visit 1 (patient screening and enrollment), Visit 2 (1-month follow-up), and Visit 3 (3-month follow-up and study completion). Between these visits, patients took the device home and were instructed to use the TAPS therapy twice daily (Figure 1A). The study protocol was approved by Institutional Review Boards for each participating site, and informed consent was obtained from each patient.

To be eligible for this study, patients had to have been previously diagnosed with ET by a physician, be ≥22 years of age, have at least one dominant hand task scoring ≥2 on the clinician-rated Tremor Research Group Essential Tremor Rating Assessment Scale (TETRAS) [32] and ≥3 on the self-rated Bain & Findley Activities of Daily Living (BF-ADL) [33], and have a total score across all dominant hand tasks ≥6 on TETRAS and ≥8 on BF-ADL. The six TETRAS dominant hand tasks assessed were (1) forward outstretched postural, (2) lateral postural, (3) kinetic, (4) spiral, (5) handwriting, and (6) dot approximation, with each task rated on a scale of 0 (“no tremor”), 1 (“slight, barely noticeable tremor; <0.5 cm amplitude”), 2 (“mild, obvious tremor; <3 cm”), 3 (“moderate, portions of drawing or writing not legible; <10 cm”), to 4 (“severe, drawing or writing complete illegible; ≥10 cm”) [32]. The eight BF-ADL dominant hand tasks assessed were (1) use a spoon to drink soup, (2) hold a cup of tea, (3) pour milk from a bottle, (4) dial a telephone, (5) pick up change, (6) insert an electric plug, (7) unlock front door, and (8) write a letter, with each task performed using in-office props and rated on a scale of 1 (“able to do without difficulty”), 2 (“able to do with little effort”), 3 (“able to do with a lot of effort”), to 4 (“cannot do without assistance”) [33]. If a patient was on medication to treat tremor, medication dosage had to be unchanged for at least 30 days prior to enrollment. Exclusion criteria included prior DBS, prior thalamotomy, epilepsy, skin lesions or eruptions at the targeted stimulation site, neuropathy of the tested upper extremity, any neurodegenerative disease aside from tremor, use of botulinum toxin for treatment of hand tremor within six months of enrollment, pregnancy, and significant alcohol or caffeine intake within 8 hours before enrollment.

### Device description, calibration, and usage

Patients were treated with a wrist-worn TAPS neuromodulation device that consisted of an electrical stimulator and a detachable band with two working electrodes positioned over the median and radial nerves and a counter-electrode positioned on the dorsal side of the wrist (Figure 1B). For each TAPS therapy session, the device electrically stimulated the median and radial nerves for 40 minutes with an alternating bursting pattern tuned to the frequency of each patient’s tremor (details below) [29]. The device included an onboard accelerometer to measure tremor physiology, and a base station that charged the device and streamed the device data to a centralized study database.

At Visit 1, study personnel fitted patients with a small, medium, or large band according to the patient’s wrist circumference, and helped patients set up the device. To calibrate the device’s bursting frequency, patients performed a series of 20-second postural holds to measure their tremor frequency (Figure 1C). For this calibration, patients performed either a forward outstretched or lateral postural hold, based on whichever was more severe. To set the stimulation intensity, study personnel gradually increased the stimulation until the patient reported paresthesia in the hand and fingers corresponding to the distribution of the median and radial nerves. The stimulation was then further increased to the maximum level that caused no discomfort or muscle contraction. Thereafter, at the start of each therapy session, the device ramped to this stimulation level and provided therapy for 40 minutes. Patients had the option to adjust the stimulation level at any time.

Patients received therapy sessions at each of the three in-clinic visits and were instructed to use the device at home twice daily for three months. They were instructed to perform the home therapy sessions at least 2 hours apart and to refrain from alcohol, caffeine, and device usage for at least 8 hours prior to the in-clinic visits. Immediately prior to and following each therapy session, the device prompted users to perform the postural hold used to calibrate the device at Visit 1 for 20 seconds, and the device’s accelerometer measured the tremor. Additionally, immediately prior to and following each of the three in-clinic therapy sessions, a clinician rated patients on TETRAS task performance and patients self-rated BF-ADL task performance using available in-office props.

### Co-primary endpoint analyses

The pre-specified co-primary efficacy endpoints were improvement in total: (1) clinician-rated TETRAS dominant hand score, and (2) patient-rated BF-ADL dominant hand score. For each scale, the improvement was defined as the difference between the pre-stimulation score at Visit 1 and the post-stimulation score at Visit 3. The co-primary endpoints were analyzed for all patients who completed their third in-clinic visit.

The TETRAS dominant hand scores were further classified consistently with the 0 to 4-point TETRAS scale as either ‘No tremor’ (total score of 0), ‘Slight’ (1–6), ‘Mild’ (7–12), ‘Moderate’ (13–18), or ‘Severe’ (19–24). These categories correspond to having an average TETRAS score across the six assessed tasks of 0 (‘no tremor’), >0–1 (‘Slight’), >1–2 (‘Mild’), >2–3 (‘Moderate’), and >3–4 (‘Severe’). Similarly, the BF-ADL dominant hand scores were classified as either ‘No tremor’ (total score of 8), ‘Mild’ (9–16), ‘Moderate’ (17–24), or ‘Severe’ (25–32), which correspond to having an average score across the eight assessed tasks of 1 (“able to do without difficulty), >1–2 (“able to do with little effort”), >2–3 (“able to do with a lot of effort”), and >3–4 (“cannot do without assistance”) on the 1 to 4-point ADL scale.

Changes in each of the following were tested with a 2-sided t-test: (1) the co-primary endpoints, (2) TETRAS and BF-ADL scores from pre- to post-stimulation at each of the three in-clinic visits, and (3) TETRAS and BF-ADL scores from pre-stimulation at Visit 1 to pre-stimulation at Visit 3. Changes in severity classifications (i.e., ‘Mild’ – ‘Severe’) from the Visit 1 pre-stimulation to Visit 3 post-stimulation assessments were summarized as the percentage of patients in each category. Total and per-task TETRAS and BF-ADL scores were summarized using measures of central tendency and variance.

### Secondary endpoint analysis

The secondary efficacy endpoint was defined as the improvement in tremor power between the pre- and post-stimulation postural holds, as measured by the device’s accelerometer. During each 20-second postural hold, wrist acceleration data were collected at a sampling frequency of 104 Hz. The first and last 4 seconds of these data were excluded to avoid transitions in and out of the postures. The algorithm to compute tremor power included six steps: (1) separating the remaining 12-second signal into five 2.4-second nonoverlapping segments, (2) computing the power spectral density (PSD) for each segment using a fast Fourier transform (scipy.org, fft) with a 256-sample Hann window, (3) identifying frequency of the peak tremor power in the 4–12 Hz band typically associated with ET, (4) computing the integral of the PSD for each of the three accelerometer axes in the ±1.2 Hz frequency window centered on frequency identified in step 3, (5) summing over the three axes, and (6) averaging these results over the segments.

The change in each patient’s pre- and post-stimulation tremor power was defined as the median change over all valid stimulation sessions. Valid sessions were defined as all sessions with a complete 40-minutes of stimulation, pre- and post-stimulation measurement occurring within 15 minutes of the stimulation start or end, and at least 2 hours of time elapsed since the previous session. A Wilcoxon signed-rank test was used to test for a change from the pre- to post-stimulation tremor power.

The improvement ratio for each patient was defined as the median of the ratios of pre- to post-stimulation tremor power over all valid sessions. With this definition, an improvement ratio of 1 indicates that tremor power was unchanged from pre- to post-stimulation, a ratio >1 indicates that tremor power improved (i.e., decreased) from pre- to post-stimulation, and a ratio <1 indicates that tremor power worsened (i.e., increased) from pre- to post-stimulation.

To compare the clinical TETRAS tremor severity ratings with the objective physiologic measurements of tremor power, at the first clinic visit patients performed three postural holds during which the device measured wrist acceleration and clinicians simultaneously provided TETRAS ratings. The association between the average TETRAS rating and the log_10_-transformed average tremor power was quantified using the Pearson correlation coefficient [34].

### Safety endpoints

Device safety was evaluated by the incidence of device- and therapy-related adverse events (AEs). These data were summarized using frequency counts and percentages.

### Exploratory analyses

Exploratory analyses included evaluating Clinical and Patient Global Impression of Improvement (CGI-I, PGI-I, respectively) scores, assessed at the study’s conclusion, and change in the average domain score of Quality of Life in Essential Tremor Questionnaire (QUEST) from the start to conclusion of the study [35,36,37]. CGI-I and PGI-I scores were summarized using response percentages, and QUEST was tested for change using a 2-sided t-test.

To assess the effect of concurrent ET medication usage on treatment efficacy, the statistical comparisons evaluating co-primary and secondary endpoints were repeated for the on-medication and off-medication patient sub-groups.

To assess device usability, patients were asked to complete a product survey rating convenience and ease-of-use of the device. To assess the duration of therapeutic effect, patients were asked “Did tremor relief last after a stimulation dose?” and, if they answered yes, were asked “On average, how long did tremor relief last after a stimulation dose?”.

### Significance testing

All reported p-values have been adjusted using Holm-Bonferroni corrections [38] for multiple comparisons. Significance for all analyses was set at p < 0.05 after corrections. Unless otherwise specified, outcome statistics are reported as mean ± 1 standard error.

## Results

### Study enrollment and completion

The study enrolled 263 patients across 26 sites (Table 1). 205 of the 263 enrolled patients completed their third in-clinic visit and were included in the primary endpoint analysis. Discontinuations included withdrawal of consent (n = 27), adverse events (n = 8), investigator decision (n = 1), failure to complete Visit 3 procedures (n = 9), and other reasons (n = 13). Reasons cited for withdrawal from the study included time commitment, lack of benefit, device malfunctions, fear of AE reoccurrence, falling out of eligibility criteria, dislike of stimulation sensation, and other or unspecified reasons.

**Table 1:** Enrolled patient demographics (N = 263).

Demographics	

**Female**	52% (137)
**Age**	69.6 ± 10.1 (23–89)
**BMI**	28.2 ± 5.4 (16–48)
**Race**	
Asian	4% (11)
Black or African American	3% (7)
White	90% (237)
More than one race	1% (3)
Unknown or not reported	2% (5)
**Ethnicity**	
Hispanic or Latino	3% (7)
Not Hispanic or Latino	96% (253)
Unknown or not reported	1% (3)
**Clinical Characteristics**	

**Onset Age**	43.9 ± 20.4 (2–79)
**ET Duration**	25.6 ± 18.1 (1–76)
**Family History**	
Yes	62% (163)
No	27% (71)
Don’t Know	11% (29)
**On ET Medications**	66% (173)
**On Antidepressant Medications**	14% (36)
**Prior ET Treatment (Any)**	78% (206)
**Prior ET Medications**	78% (205)
**Prior Botulinum**	4% (11)
**Responsive to Alcohol**	37% (96)

Reported as % patients (#) or mean ± SD (min – max).

On average, these 205 patients completed at least one stimulation session per day for 78% of the days they were enrolled in the study and completed 68% of their total instructed (i.e., twice-daily) stimulation sessions (see Supplemental Figure 1 for distribution of stimulation session adherence). 193 of these 205 patients completed a total of 21,806 valid stimulation sessions at home and were included in the secondary endpoint analysis. 10 patients were excluded due to errors with the accelerometer recordings, 2 patients were excluded due to incorrect device calibration, and 1,808 stimulation sessions from the remaining 193 patients were excluded due to missing valid pre- and/or post-stimulation measurements.

### Co-primary outcomes

**Figure 2:**
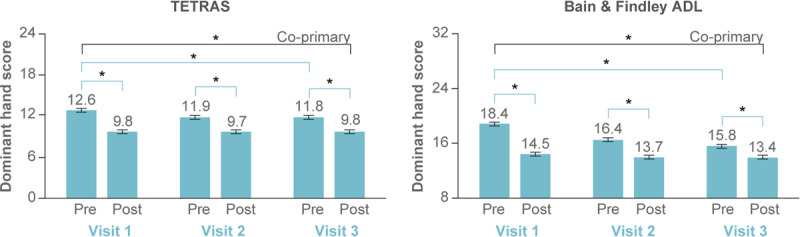
**Co-primary endpoints assessed in-clinic showed improvement in TETRAS and BF-ADL.** Average TETRAS dominant hand score (left, scale range 0–24) and BF-ADL dominant hand score (right, scale range 8 to 32) are shown pre- and post-stimulation conducted at each in-clinic visit. The co-primary TETRAS and BF-ADL endpoints—improvement from baseline (pre-stimulation rating at Visit 1) to study exit (post-stimulation rating at Visit 3)—were both met (n = 205). Therapeutic response was also significant within each visit for both TETRAS and BF-ADL, and the pre-stimulation tremor rating improved significantly over 3 months of use. Error bars represent ±1 SEM, and * indicates p < 0.0001.

TETRAS and BF-ADL dominant hand scores improved from baseline to study exit (i.e., Visit 1 pre-stimulation to Visit 3 post-stimulation; Table 2, Figure 2). Patients showed improvement in TETRAS and BF-ADL from pre- to post-stimulation at each in-clinic visit (p ≪ 0.0001 for all six pairs; Figure 2). Additionally, pre-stimulation tremor level improved from Visit 1 to Visit 3 on both TETRAS and BF-ADL (p ≪ 0.0001 for both) (Figure 2).

**Table 2:** Descriptive statistics of co-primary and secondary endpoints.

	Baseline Mean (SD)	Final visit Mean (SD)	Change Mean (SD)

**Co-primary endpoints**^1^				
TETRAS dominant hand score^2^	12.6 (2.7)	9.8 (3.5)	–2.8 (2.8)*
BF-ADL dominant hand score^3^	18.4 (3.8)	13.4 (4.4)	–5.0 (4.3)*
**Secondary endpoint**^4^				
Tremor power (m/s^2^)^2^	1.1 (4.4)	0.3 (1.1)	–0.8 (3.7)*

* p ≪ 0.0001 after Holm-Bonferroni corrections for multiple hypothesis testing. ^1^ n = 205; ^2^ Minimum score 0, maximum score 24; ^3^ Minimum score 8, maximum score 32; ^4^ n = 193.

**Figure 3:**
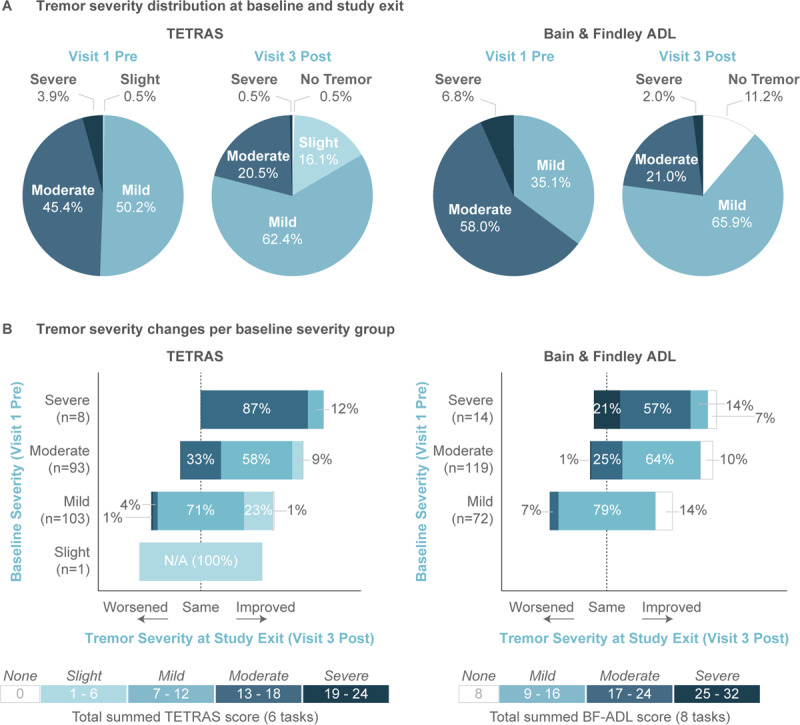
**Tremor severity distributions shifted towards milder tremor at study exit. (A)** The distribution of tremor severity at the time of co-primary endpoints (i.e., Visit 1 pre-stimulation to Visit 3 post-stimulation) were assessed for TETRAS (left) and BF-ADL (right). On both scales, the distribution of tremor severity shifted towards milder tremor. **(B)** Study exit (Visit 3 post-stimulation) tremor severity distributions were broken down for each baseline severity group for TETRAS (left) and BF-ADL (right). Most patients improved in tremor severity relative to their baseline or stayed in the same severity classification, with more severe patients showing greater improvement. Severity categories were defined consistent with TETRAS guidelines.

The proportion of patients rated “Severe” or “Moderate” improved from 49.3% (TETRAS) and 64.8% (BF-ADL) at baseline (Visit 1 pre-stimulation) to 21.0% (TETRAS) and 23.0% (BF-ADL) at study exit (Visit 3 post-stimulation; Figure 3A). While the magnitude of improvement varied between patients (see Supplemental Figure 2 for distribution of TETRAS and BF-ADL improvements), 62% of patients with a “Severe” or “Moderate” TETRAS score (score between 13 and 24) improved to “Mild” or better (score ≤ 12), and 68% of patients with a “Severe” or “Moderate” BF-ADL score improved to “Mild” or better (score ≤ 16; Figure 3B). Only a small number of patients worsened in severity category (5 for TETRAS, 6 for BF-ADL; Figure 3B) or improved in severity category with a ≤1-point change in score (3 for TETRAS, 1 for BF-ADL).

Per-task improvements from baseline to study exit showed that on any rated task, between 58%–80% (TETRAS) and 61%–76% (BF-ADL) of patients who were rated as at least “Mild” improved at least one rating-increment on the task’s scale (Table 3). Per-task improvements were variable and responder rates were lower (between 45%–74%) among the full study population due to ceiling effects on improvement of patients scoring below “Mild” on each task (Supplemental Table 1; right columns).

**Table 3:** Co-primary outcomes by task.

	Patient count per task^1^	Baseline Mean (SD)	Final visit Mean (SD)	Change Mean (SD)	% Patients improved^2^

**TETRAS Tasks**^3^						
Forward Outstretched	124	2.2 (0.3)	1.5 (0.6)	–0.6 (0.6)*	78%
Lateral	140	2.3 (0.4)	1.7 (0.7)	–0.6 (0.6)*	80%
Kinetic	163	2.3 (0.4)	1.7 (0.6)	–0.6 (0.5)*	79%
Spiral	161	2.5 (0.7)	2.0 (0.8)	–0.5 (0.8)*	58%
Handwriting	144	2.8 (0.7)	2.0 (1.0)	–0.8 (0.8)*	67%
Dot Approximation	155	2.4 (0.5)	1.9 (0.7)	–0.4 (0.6)*	66%
**BF-ADL Tasks**^4^						
Use a spoon to drink soup	196	2.9 (0.6)	2.0 (0.9)	–0.9 (0.8)*	70%
Hold a cup of tea	192	2.8 (0.7)	1.8 (0.9)	–1.0 (0.9)*	71%
Pour milk from a bottle	182	2.8 (0.7)	1.8 (0.9)	–1.0 (0.9)*	69%
Dial a telephone	131	2.6 (0.7)	1.8 (0.9)	–0.8 (0.8)*	76%
Pick up change	134	2.6 (0.7)	1.8 (0.9)	–0.8 (0.8)*	69%
Insert an electric plug	134	2.4 (0.5)	1.5 (0.6)	–0.9 (0.8)*	69%
Unlock front door	148	2.4 (0.5)	1.5 (0.6)	–0.9 (0.8)*	72%
Write a letter	192	2.3 (0.5)	1.5 (0.7)	–0.8 (0.8)*	61%

*p ≪ 0.0001 after Holm-Bonferroni corrections for multiple hypothesis testing. ^1^ Count of patients scoring at least “Mild” per task (2 on TETRAS or BF-ADL). ^2^ Defined as % patients improving at least one increment (0.5 or 1, depending on scale and task). ^3^ Each TETRAS task rated 0–4 by clinician (0 = normal, 1 = slight, 2 = mild, 3 = moderate, 4 = severe). ^4^ Each BF-ADL task rated 1–4 by patient (1 = without difficulty, 2 = with a little effort, 3 = with a lot of effort, 4 = cannot do by yourself).

### Secondary outcomes

**Figure 4:**
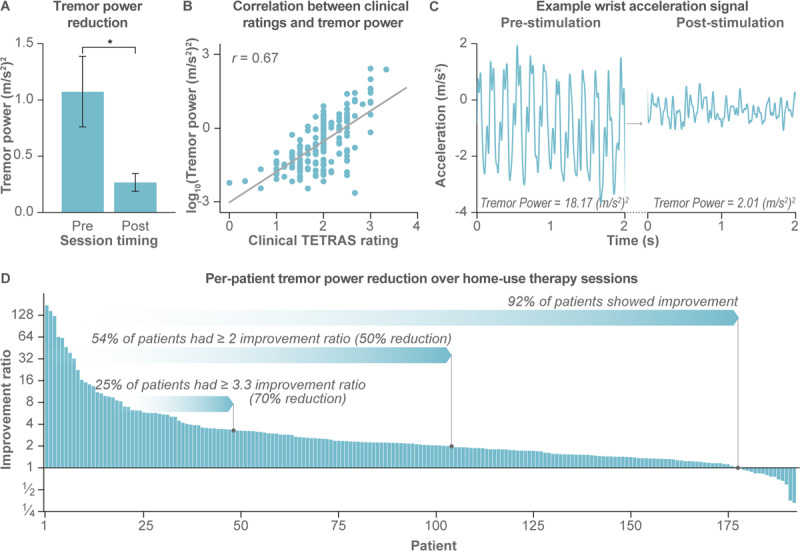
**Secondary endpoint from at-home accelerometer measures show improvement in tremor physiology with therapy. (A)** Average tremor power decreased from pre-stimulation to post-stimulation (data represents 193 patients and 21,806 total sessions). Error bars represent ±1 SEM, and * indicates p < 0.0001. **(B)** Tremor power, computed from the triaxial acceleration signals, was correlated to the clinician-rated TETRAS postural hold rating (r = 0.67, p < 0.0001). **(C)** Example 3-second segment of the wrist acceleration time series along one of the three accelerometer axes with corresponding tremor power measures are shown for a single session before and after stimulation. **(D)** 92% of all patients had an improvement ratio > 1, indicating an improvement in tremor power from pre- to post-stimulation. Each bar represents a single-patient’s median improvement in tremor power from pre- to post-stimulation over all at-home stimulation sessions over three months (n = 193 patients).

Tremor power improved during home use, with the mean tremor power over all patients decreasing from 1.1 ± 0.3 (m/s^2^)^2^ pre-stimulation to 0.3 ± 0.1 (m/s^2^)^2^ post-stimulation (p ≪ 0.0001) (Table 2; Figure 4A). The log_10_-tremor power was correlated to the simultaneously measured TETRAS ratings (r = 0.67, p ≪ 0.0001) (Figure 4B), with equation (1) describing the mathematical relationship.

1 {\log _{10}}\left({{\rm{Tremor\ Power}}} \right)\ =\ 1.26\ \cdot \ {\rm{TETRAS}}\ -\ 3.13

A sample raw acceleration trace corresponding to a 9-fold reduction (i.e., strong therapeutic response) in tremor power from pre- to post-stimulation is shown for illustrative purposes (Figure 4C). Overall, daily usage of the device resulted in a median improvement in tremor power over all stimulation sessions for 92% of patients (Figure 4D). 54% of patients had a ≥2 improvement ratio in tremor power (i.e., post-tremor power ≤½ pre-tremor power, or 50% reduction in pre-tremor power), and 25% of patients had a ≥3.3 improvement ratio (70% reduction) in tremor power.

### Safety outcomes

No device-related serious AEs were reported. Non-serious device-related AEs occurred in 18% patients. The most common device-related AEs were persistent skin irritation (5% patients), sore/lesion (4% patients), discomfort (2% patients), electrical burns (2% patients), and minor skin irritation including itchiness or redness (2% patients) (Table 4). 64% of the reported device-related AEs were rated by the clinical investigator as “Mild” (e.g., itchiness, discomfort), 34% as “Moderate” (e.g., electrical burns, significant discomfort), and 2% (1 event) as “Severe” (a fall, that was possibly device-related). All device-related AEs were resolved either without intervention, with decreasing stimulation amplitude, with a topical ointment such as aloe vera or hydrocortisone cream, or by discontinuing therapy until resolved. There was only one report of minor sequelae that occurred in a patient with pre-existing psoriasis. There were 6 withdrawals due to device-related AEs, of which 3 were due to skin irritation and 3 were due to discomfort, anxiety, and tremor worsening.

**Table 4:** Device-related adverse events.

Adverse Event Type^1^	% Subjects (#)	# Events

**All**	**17.9% (47)**	**56**
Significant and persistent skin irritation (including redness, itchiness, and/or swelling)	5.3% (14)	15
Sore/Lesion	3.8% (10)	11
Significant discomfort	2.3% (6)	7
Electrical burns	2.3% (6)	6
Other: minor skin irritation (including itchiness and/or redness)	2.3% (6)	6
Other: electric shock sensation while using device	1.1% (3)	3
Other: worsening of tremor	0.8% (2)	2
Other isolated events^2^	2.0% (5)	6

^1^ Rated by investigator to be possibly, probably or definitely device-related. ^2^ Each of the following occurred in only one patient: fall, anxiety, intermittent soreness in treated wrist, weakness or lack of coordination in treated hand, persistent pain from stimulation.

### Exploratory outcomes

**Figure 5:**
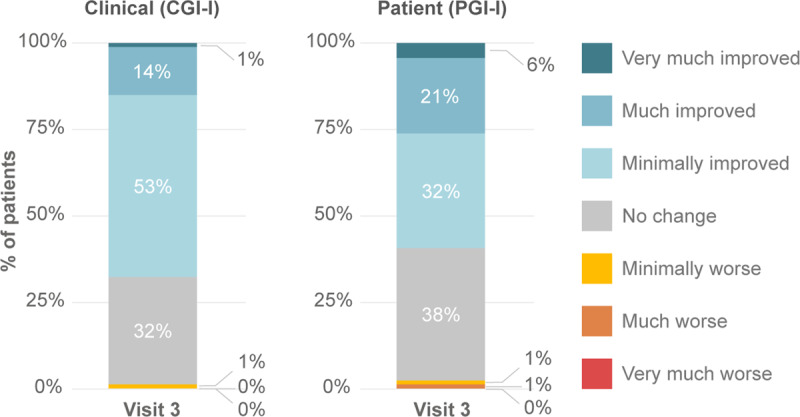
**Clinical and Patient Global impression of improvement (C/PGI-I).** Clinicians and patients were surveyed with the 7-point global impression scale of improvement at Visit 3 post the in-clinic stimulation session to assess improvements in dominant hand tremor relative to baseline. Clinicians (CGI-I) and patients (PGI-I) reported hand tremor minimally, much or very much improved in 68% and 60% of patients, respectively.

After three months of use, clinicians reported tremor improvement in 68% of patients (15% much improved or very much improved; CGI-I) and 60% of patients self-reported improvement (27% much improved or very much improved; PGI-I) (Figure 5). In QUEST surveys conducted after three months of use, patients indicated their quality of life improved (–3.1 ± 0.9 change in QUEST average domain score, p = 0.0019). Among the QUEST domains, physical domain improved the most (–6.3 ± 1.2, p ≪ 0.0001), followed by work and finance domains (–3.6 ± 1.1, p = 0.0015).

The therapy was effective for patients, regardless of concurrent ET medication usage. Patients off ET medication (n = 66) improved by 3.2 ± 0.3 points on TETRAS (p ≪ 0.0001) and 5.3 ± 0.5 points on BF-ADL (p ≪ 0.0001) from pre-stimulation Visit 1 to post-stimulation Visit 3. Patients on ET medication (n = 139) improved by 2.6 ± 0.2 points on TETRAS (p ≪ 0.0001) and 4.8 ± 0.4 points on BF-ADL (p ≪ 0.0001) from pre-stimulation Visit 1 to post-stimulation Visit 3. Similarly, tremor power decreased from 1.40 ± 0.74 pre-stimulation to 0.25 ± 0.10 post-stimulation (p ≪ 0.0001) for patients off medication (n = 65), and from 0.91 ± 0.30 to 0.28 ± 0.11 (p ≪ 0.0001) for patients on medication (n = 128). The improvements in TETRAS, BF-ADL, and tremor power were not statistically different between the patients off- and on-medication.

On patient surveys, 85% of patients reported that the device was convenient and easy to use, and 64% of patients reported persistent tremor relief after the 40 minutes of stimulation lasting on average 94 minutes (standard deviation = 138; median = 60).

## Discussion

This study suggested that TAPS therapy provided repeatable therapeutic benefit with a favorable safety profile over three months of use in adults with ET. Despite the heterogeneity of ET presentation, the day-to-day symptomatic variability of the disorder, and the variable therapeutic needs of individual patients, the therapeutic response was reproduced across multiple acute and longitudinal improvement measures, including clinician-rated TETRAS and CGI-I scores, patient-rated BF-ADL, PGI-I, and quality of life scores (Figures 2, 3 and 5), and objective accelerometer-measured tremor power improvements (Figure 4).

The reductions in tremor and the absence of serious device-related adverse events suggest that TAPS is a safe and effective therapy option for ET. Over 50% of patients had a ≥2-fold reduction in tremor power with daily TAPS therapy (Figure 4D) and, for most (64%) patients, tremor relief endured on average 90+ minutes following the therapy session. These tremor reductions are comparable to reductions obtained with first-line pharmacotherapies propranolol and primidone [17,18]. While ET medications are effective in approximately half of patients, their side effects at the doses required to reduce tremor cause many patients to discontinue use [17,18,20,21,22]. This study did not find a relationship between concurrent ET medication usage and response to TAPS therapy, but future work is needed to better understand the underlying patient characteristics and interactions between multiple therapeutic approaches. Further, around one in four patients in our study experienced tremor reduction similar to the 55–90% tremor reduction reported for invasive surgical therapies including DBS and MRgFUS [17,23]. Though highly effective, DBS poses a risk of serious adverse events, can lead to dysarthria and dysphagia, and for some patients can lose efficacy over time [23,39,40]. Advanced age, cognitive impairment, and other health issues can limit access to DBS [41,42], and some patients discontinue DBS therapy due to VIM DBS-related side effects [41]. MRgFUS also carries risks of side effects, with gait ataxia, unsteadiness, and hand ataxia as the most commonly reported AEs [23]. In a small number of patients, these side-effects were found to be irreversible. The TAPS therapy tested in this study was devoid of device-related serious AEs, and all AEs were reversible with small changes (e.g., lowering device stimulation level or topical, over-the-counter ointment) or no intervention, differentiating it from surgical and pharmacological treatments.

This study also demonstrated the benefits of adding objective at-home accelerometer-based measure of tremor physiology to standard in-clinic assessments. Consistent with previous reports of sensor-based measurements [43,44,45], this study’s accelerometer-based measurements of tremor power were correlated with gold-standard clinician-ratings (Figure 4B). While the improvements in TETRAS and BF-ADL scores quantified treatment efficacy for each patient at three instances over the three-month study duration, the accelerometer-based metrics quantified treatment efficacy for, on average, 113 therapy sessions per patient (21,806 sessions for 193 patients). The objectivity and frequency of these sensor-based measurements overcome key limitations of previous single-session stimulation studies [28,29]. These data demonstrate how wearable technologies can enable out-of-clinic remote monitoring of tremor and can be used to identify whether a treatment remains effective over longitudinal use [44,45]. Future work that expands remote tremor physiology assessment to remove the inconvenience of performing postural holds and to develop metrics that quantify functional ability throughout the day would benefit the field.

This study had a few important limitations that should be considered while interpreting its results. First, the open-label, single-arm design limits conclusions reliant on assessment of longitudinal repeated-use sham response. A previous 23-patient blinded, randomized single-session trial using an earlier version of TAPS therapy showed that TETRAS spiral drawing scores had greater improvements with TAPS therapy compared to sham [28]. A similarly constructed multi-site trial with 77 patients did not reproduce this spiral drawing finding, but found that TAPS therapy resulted in greater improvements compared to sham in the TETRAS scores summed for a lateral postural hold, forward outstretched postural hold, and kinetic finger-nose-finger testing, and improvements in tremor amplitude [29]. However, the latter study’s blinding index of 0.608 [46] suggested it would be challenging to successfully maintain a blind over months of at-home usage. An active sham with altered parameters such as a different stimulation bursting frequency or vibrotactile sensory stimulation could be considered; however, such designs risk activating neural circuitry via alternate pathways and may not provide a true, treatment-free control. Future research to establish robust methods to longitudinally maintain a patient blind for peripheral neuromodulation therapies would be a valuable asset for assessing novel therapies.

A sham arm could have also controlled for any improvements due to learning effects as patients grew more comfortable with performing the various tremor tasks. For example, this study found pre-stimulation TETRAS and BF-ADL ratings at Visit 3 were lower than pre-stimulation ratings at Visit 1, which may be partially attributable to learning effects. A post-hoc secondary endpoint analysis that segmented the at-home data into the first, second, and third months of the trial found that acute therapeutic efficacy was similar over time (median improvement ratios of 2.0 in month 1, 2.3 in month 2; and 2.0 in month 3), and substantially greater than the improvement in median pre-stimulation tremor power from month 1 to month 3 (improvement ratio of 1.1). The consistency of response over the three months at home suggests a reproducible therapeutic effect even with task-learning effects. It is possible the cumulative reduction in baseline tremor severity may also be partially attributable to neurophysiological remodeling resulting from repeated use of TAPS therapy. Future studies on longitudinal mechanisms of action of this therapy could be valuable to understand this contribution.

Second, clinical raters were unblinded to the study’s design, which may have introduced bias into the TETRAS ratings, e.g., from pre- to post-stimulation at each of the three in-clinic visits. Encouragingly, the objective tremor measurements at the in-clinic visits showed that tremor power decreased with stimulation (median improvement ratio of 1.7 at Visits 1 and 3) and that this decrease was directionally consistent with reductions in clinical TETRAS ratings (Figure 4D). The confounding effect of rater-bias could be addressed by using central ratings blinded to the study timepoints. While TETRAS rating by video has been validated [47] and successfully used in some acute studies evaluating ET therapies [28,29], a recent study on non-invasive pharmacologic therapy suggested methodological concerns with central ratings [48].

Third, while the study found statistically significant reductions across all tremor subtasks in both the TETRAS and BF-ADL ratings, in part due to the study’s unprecedented sample size, the magnitude of those reductions varied between tasks (Table 3, Supplemental Table 1). Across tasks, there were 20–40% of patients for whom TAPS therapy did not relieve specific tremor symptoms. We expect there are two main reasons driving the observed variability in individual and population-level response. Latent patient subtypes may influence the variable treatment response observed with all current ET therapies (i.e., pharmacotherapy, invasive therapy (DBS, MRgFUS), and non-invasive TAPS therapy). While there is general consensus on the existence of these subtypes [49] (e.g., early-onset vs late-onset ET), the full range of sub-types, their clinical presentation, and their interaction with therapeutic interventions has not been fully characterized [50].

Similarly, patients in this study had diverse symptomatic presentations of tremor. We do not expect TAPS therapy to improve tremor rating in a task that did not elicit tremor for that patient, which creates a ceiling on maximum improvement for that patient and accordingly lowers population-level average improvements. To our knowledge there are no defined standards for what constitutes a clinically meaningful improvement in TETRAS or BF-ADL, though the resolution of the scales (0.5 or 1 point, depending on the scale and task) [32,33] and the community characterization of intra- and inter-rater reliability for these scales [51,52] suggests that minimum detectable improvement thresholds defined by the scale’s resolution can be considered clinically meaningful. Encouragingly, tremor improvements in this study were larger and consistently on the order of the task-specific minimum detectable improvements for the subsets of patient who had baseline tremor (i.e., at least a “Mild” tremor) in a given subtask (Table 3).

Finally, the pre-specified primary and secondary endpoints in this study excluded the fifty-eight patients who exited the study early and therefore did not qualify for the pre-specified analyses, which may have biased the study’s reported responder rates. Fourteen of these 58 patients cited “lack of device benefit” as the reason for withdrawal of consent. A worst-case analysis treating these 14 patients as “non-responders” would lower this study’s reported responder rates by less than 5%. However, a post-hoc analysis found improvements in TETRAS and BF-ADL were not statistically different between those that completed the study, those withdrew citing lack of benefit, and those that withdrew citing other reasons (e.g., adverse events, time commitment; Supplemental Figure 3A). Likewise, these patients’ median at-home improvement ratios were comparable (Supplemental Figure 3B). The similarity in response across these three patient cohorts suggests that the study reflected the expected range of therapeutic responses in the ET patient population; and the variability in patient perception despite the similar measured response profiles highlights opportunities for the field to continue developing patient-centered metrics of meaningful therapeutic improvement.

In conclusion, this study suggests that TAPS therapy is safe and improves hand tremor and quality of life over three months of use in a large cohort of patients with ET. Future work examining how these clinical trial results translate into the real-world setting would be valuable.

## Additional Files

The additional files for this article can be found as follows:

10.5334/tohm.59.s1 Supplemental Figure 1. Distribution of adherence to prescribed sessions.

10.5334/tohm.59.s2 Supplemental Figure 2. Distribution of co-primary tremor rating improvements.

10.5334/tohm.59.s3 Supplemental Figure 3. Comparison between patients who completed study (n = 205) and patients who withdrew citing lack benefit (n = 14) or other reasons (n = 44).

10.5334/tohm.59.s4 Supplemental Table 1. Co-primary outcomes by task for full study population compared to patient subgroups with at least mild tremor power task.

## Figures and Tables

**Table:** 

Name	Location	Role	Contribution

Stuart Isaacson, MD	Parkinson’s Disease and Movement Disorders of Boca Raton, Boca Raton	Author	Study design, Principal investigator on the research, manuscript review and critique
Elizabeth Peckham, DO	Central Texas Neurology Consultants, Round Rock	Author	Site investigator on the research, manuscript review and critique
Winona Tse, MD	Mount Sinai Hospital, New York	Author	Site investigator on the research, manuscript review and critique
Olga Waln, MD	Houston Methodist, Houston	Author	Site investigator on the research, manuscript review and critique
Christopher Way, DO	Parkinson’s Institute and Clinical Center, Mountain View	Author	Site investigator on the research, manuscript review and critique
Melita Petrossian, MD	Pacific Neuroscience Institute, Santa Monica	Author	Site investigator on the research, manuscript review and critique
Nabila Dahodwala, MD, MSc	University of Pennsylvania. Philadelphia	Author	Site investigator on the research, manuscript review and critique
Michael Soileau, MD	Texas Movement Disorders Specialists, Georgetown	Author	Site investigator on the research, manuscript review and critique
Mark Lew, MD	USC, Los Angeles	Author	Site investigator on the research, manuscript review and critique
Cameron Dietiker, MD	UCSF, San Francisco	Author	Site investigator on the research, manuscript review and critique
Nijee Luthra, MD	UCSF, San Francisco	Author	Site investigator on the research, manuscript review and critique
Pinky Agarwal, MD, FAAN	EvergreenHealth, Kirkland	Author	Site investigator on the research, manuscript review and critique
Rohit Dhall, MD, MSPH	UAMS, Little Rock	Author	Site investigator on the research, manuscript review and critique
John Morgan, MD, PhD	Augusta University, Augusta	Author	Site investigator on the research, manuscript review and critique
Nicole Calakos, MD, PhD	Duke University, Durham	Author	Site investigator on the research, manuscript review and critique
Theresa Zesiewicz, MD	USF, Tampa	Author	Site investigator on the research, manuscript review and critique
Ejaz A. Shamim, MD, MS, MBA, FAAN	Kaiser Permanente, MidAtlantic States, MidAtlantic Permanente Research Institute	Author	Site investigator on the research, manuscript review and critique
Rajeev Kumar, MD	Rocky Mountain Movement Disorders Center, Englewood	Author	Site investigator on the research, manuscript review and critique
Peter LeWitt, MD	Henry Ford Health System, West Bloomfield	Author	Site investigator on the research, manuscript review and critique
Holly Shill, MD, FAAN	Barrow Neurological Institute, Phoenix	Author	Site investigator on the research, manuscript review and critique
Adam Simmons, MD	Hospital for Special Care, New Britain	Author	Site investigator on the research, manuscript review and critique
Fernando Pagan, MD	Georgetown University, Washington DC	Author	Site investigator on the research, manuscript review and critique
Pravin Khemani, MD	Swedish, Seattle	Author	Site investigator on the research, manuscript review and critique
Jessica Tate, MD	Wake Forest, Winston-Salem	Author	Site investigator on the research, manuscript review and critique
Brian Maddux, MD	Riverhills Neuroscience, Cincinnati	Author	Site investigator on the research, manuscript review and critique
Lan Luo, MD, MS	Beth Israel Deaconess Medical Center, Boston	Author	Site investigator on the research, manuscript review and critique
William Ondo, MD	Houston Methodist, Houston	Author	Device Safety Monitoring Board; manuscript review and critique
Mark Hallett, MD	NIH, Bethesda	Author	Study design, manuscript review and critique. No clinical work was done at NIH.
Apoorva Rajagopal, PhD	Cala Health, Burlingame	Author	Manuscript drafting, review and critique
Paula Chidester, MS	Cala Health, Burlingame	Author	Study design, manuscript drafting, review and critique
Kathryn Rosenbluth, PhD	Cala Health, Burlingame	Author	Study design, manuscript drafting, review and critique
Scott Delp, PhD	Stanford University, Stanford	Author	Study design, manuscript drafting, review and critique
Rajesh Pahwa, MD	University of Kansas Medical Center, Kansas City	Author	Study design, Principal investigator on the research, manuscript review and critique
