# Supplementary material for: Prospective Home-use Study on Non-invasive Neuromodulation Therapy for Essential Tremor
Source: Tremor Other Hyperkinet Mov (N Y). 2020 Aug 14;10:29. doi: 10.5334/tohm.59 (PMC7427656; doi:10.5334/tohm.59)
Supplement: Supplemental Figure 1. — Distribution of adherence to prescribed sessions. [file tohm-10-1-59-s1.pdf]

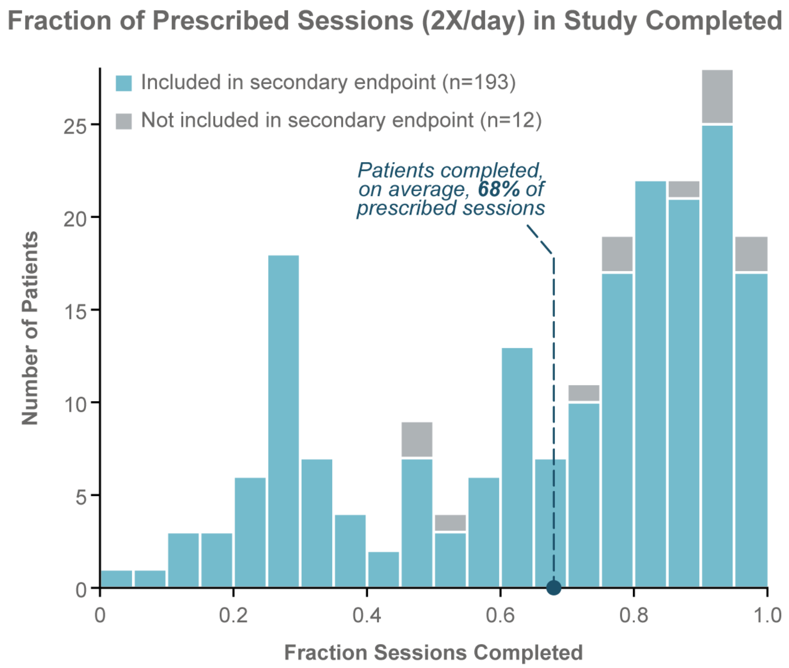

Supplemental Figure 1. Distribution of adherence to prescribed sessions. Patients were instructed to perform two stimulation sessions each day in the study. On average, patients completed 68% of the prescribed sessions. 22% of patients completed ≥ 90% of prescribed sessions; 44% of patients completed ≥ 80% of prescribed sessions; and 73% patients completed ≥ 50% of prescribed sessions.
