# Supplementary material for: Prospective Home-use Study on Non-invasive Neuromodulation Therapy for Essential Tremor
Source: Tremor Other Hyperkinet Mov (N Y). 2020 Aug 14;10:29. doi: 10.5334/tohm.59 (PMC7427656; doi:10.5334/tohm.59)
Supplement: Supplemental Figure 2. — Distribution of co-primary tremor rating improvements. [file tohm-10-1-59-s2.pdf]

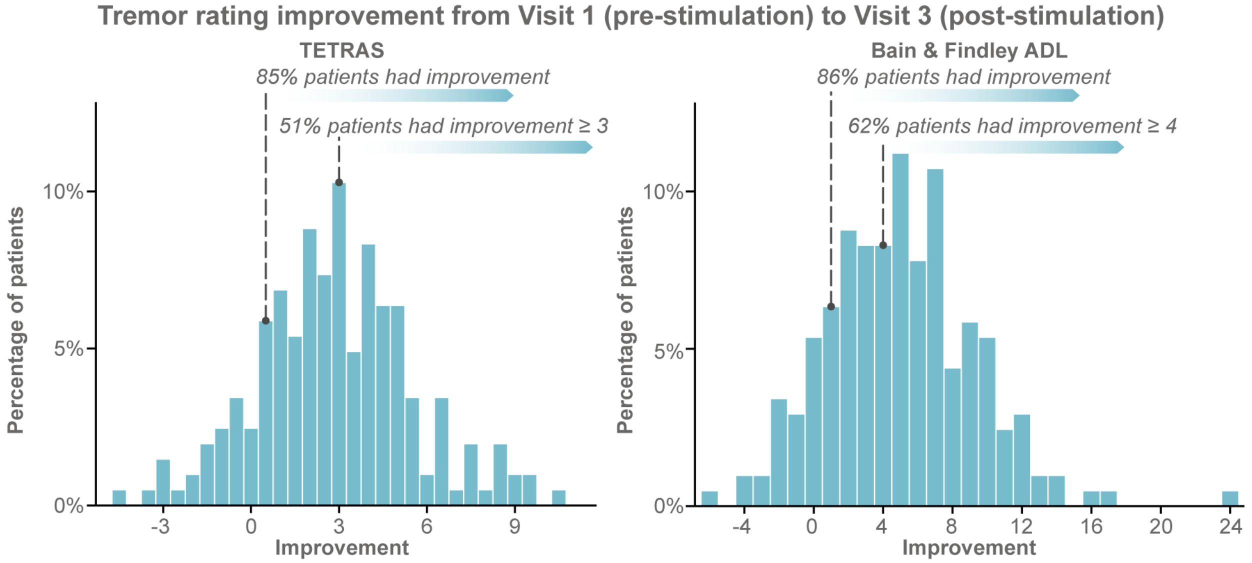

Supplemental Figure 2. Distribution of co-primary tremor rating improvements. Per-patient improvements in TETRAS (left) and BF-ADL (right) from baseline (pre-stimulation at Visit 1) to study exit (post-stimulation at Visit 3). Mean TETRAS improvement was 2.8 (95% confidence interval = [2.4, 3.2]) with standard deviation 2.8. Mean Bain & Findley ADL improvement was 5.0 (95% confidence interval = [4.4, 5.6]) with standard deviation 4.3.
