# Supplementary material for: Prospective Home-use Study on Non-invasive Neuromodulation Therapy for Essential Tremor
Source: Tremor Other Hyperkinet Mov (N Y). 2020 Aug 14;10:29. doi: 10.5334/tohm.59 (PMC7427656; doi:10.5334/tohm.59)
Supplement: Supplemental Figure 3. — Comparison between patients who completed study (n = 205) and patients who withdrew citing lack benefit (n = 14) or other reasons (n = 44). [file tohm-10-1-59-s3.pdf]

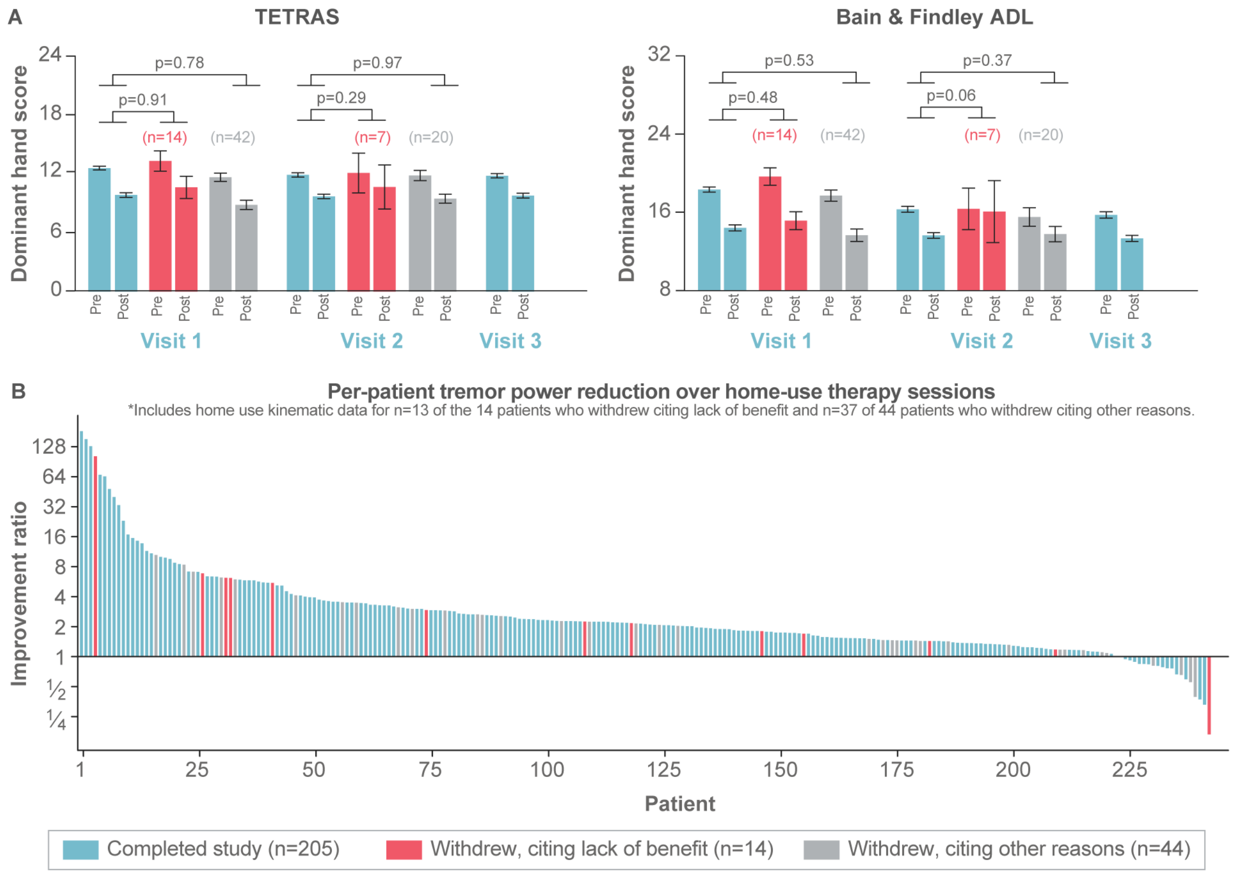

Supplemental Figure 3. Comparison between patients who completed study (n=205) and patients who withdrew citing lack benefit (n=14) or other reasons (n=44). Fourteen of the 58 patients who dropped out of the study early cited "Lack of benefit" as the reason for withdrawal of consent. These 14 patients exited the study after completing between 7 and 86 therapy sessions over 3 - 47 days in the study. (A) Improvements in TETRAS (left) and BF-ADL (right) from pre-stimulation to post-stimulation were computed at Visit 1 and Visit 2 using available data for patients who completed the study (blue), withdrew citing lack of benefit (red), and withdrew citing other reasons (gray). The improvements were not statistically different between those that completed the study and those that withdrew. Error bars represent ± 1 standard error of the mean. (B) The median improvement ratios for 50 of these 58 withdrawn patients were computed and interwoven with the cohort of patients that completed the study (8 patients were excluded either due to issue with accelerometer data or due to lack of completed, valid sessions).
