# Supplementary material for: Prospective Home-use Study on Non-invasive Neuromodulation Therapy for Essential Tremor
Source: Tremor Other Hyperkinet Mov (N Y). 2020 Aug 14;10:29. doi: 10.5334/tohm.59 (PMC7427656; doi:10.5334/tohm.59)
Supplement: Supplemental Table 1. — Co-primary outcomes by task for full study population compared to patient subgroups with at least mild tremor power task. [file tohm-10-1-59-s4.pdf]

Supplemental Table 1. Co-primary outcomes by task for full study population compared to patient subgroups with at least mild tremor power task. The left columns ("Patients with baseline score ≥ 2") are reproduced from Table 3. The added right columns ("All patients (N = 205)") illustrate how responder rates would decrease if considering all patients, including those who did not have appreciable tremor in the assessed task. This decrease is expected, as there is a ceiling effect on improvement: patients already at a score of 1 or 0 on TETRAS pre-stimulation are unlikely to or cannot improve on the TETRAS scale with any therapy; and similarly, patients at a score of 1 on BF-ADL pre-stimulation cannot improve on the BF-ADL scale with therapy.

	Patients with baseline score ≥ 2*					All patients (N = 205)			
Task	Patient count per task[1]	Baseline Mean (SD)	Final visit Mean (SD)	Change Mean (SD)	% Patients improved[2]	Baseline Mean (SD)	Final visit Mean (SD)	Change Mean (SD)	% Patients improved[2]
**TETRAS Tasks[3]**									
Forward Outstretched	124	2.2 (0.3)	1.5 (0.6)	-0.6 (0.6)*	78%	1.8 (0.5)	1.3 (0.7)	-0.5 (0.6)*	64%
Lateral	140	2.3 (0.4)	1.7 (0.7)	-0.6 (0.6)*	80%	2.0 (0.6)	1.5 (0.7)	-0.5 (0.6)*	74%
Kinetic	163	2.3 (0.4)	1.7 (0.6)	-0.6 (0.5)*	79%	2.1 (0.6)	1.6 (0.7)	-0.6 (0.6)*	73%
Spiral	161	2.5 (0.7)	2.0 (0.8)	-0.5 (0.8)*	58%	2.2 (0.9)	1.9 (0.8)	-0.3 (0.9)*	51%
Handwriting	144	2.8 (0.7)	2.0 (1.0)	-0.8 (0.8)*	67%	2.3 (1.1)	1.7 (1.1)	-0.6 (0.9)*	57%
Dot Approximation	155	2.4 (0.5)	1.9 (0.7)	-0.4 (0.6)*	66%	2.1 (0.6)	1.8 (0.7)	-0.4 (0.6)*	60%
**BF-ADL Tasks[4]**									
Use a spoon to drink soup	196	2.9 (0.6)	2.0 (0.9)	-0.9 (0.8)*	70%	2.8 (0.7)	2.0 (0.9)	-0.8 (0.9)*	67%
Hold a cup of tea	192	2.8 (0.7)	1.8 (0.9)	-1.0 (0.9)*	71%	2.7 (0.8)	1.8 (0.9)	-0.9 (0.9)*	66%
Pour milk from a bottle	182	2.8 (0.7)	1.8 (0.9)	-1.0 (0.9)*	69%	2.4 (0.8)	1.7 (0.9)	-0.7 (0.9)*	61%
Dial a telephone	131	2.6 (0.7)	1.8 (0.9)	-0.8 (0.8)*	76%	1.9 (0.8)	1.4 (0.6)	-0.5 (0.9)*	49%
Pick up change	134	2.6 (0.7)	1.8 (0.9)	-0.8 (0.8)*	69%	1.9 (0.8)	1.4 (0.6)	-0.4 (0.8)*	45%
Insert an electric plug	134	2.4 (0.5)	1.5 (0.6)	-0.9 (0.8)*	69%	1.8 (0.7)	1.4 (0.6)	-0.5 (0.8)*	45%
Unlock front door	148	2.4 (0.5)	1.5 (0.6)	-0.9 (0.8)*	72%	2.0 (0.7)	1.4 (0.6)	-0.5 (0.8)*	52%
Write a letter	192	2.3 (0.5)	1.5 (0.7)	-0.8 (0.8)*	61%	2.9 (0.8)	2.2 (0.9)	-0.7 (0.9)*	57%

*$p \ll 0.0001$ after Holm-Bonferroni corrections for multiple hypothesis testing
[1] Count of patients scoring at least "Mild" per task (2 on TETRAS or BF-ADL)
[2] Defined as % patients improving at least one increment (0.5 or 1, depending on scale and task)
[3] Each TETRAS task rated 0-4 by clinician (0 = normal, 1 = slight, 2 = mild, 3 = moderate, 4 = severe)
[4] Each BF-ADL task rated 1-4 by patient (1 = without difficulty, 2 = with a little effort, 3 = with a lot of effort, 4 = cannot do by yourself).
